# “I base my life on sadness”: Apparently paradoxical sources of resilience among young Haitians

**DOI:** 10.1177/13634615231202094

**Published:** 2023-09-27

**Authors:** Kristine Elvevold Andreassen, Anna Luise Kirkengen, May-Lill Johansen

**Affiliations:** 1 Community Medicine, 60482 UiT The Arctic University of Norway Faculty of Health Sciences, Tromsø, Norway; 2 General Practice Research Unit, Department of Public Health and Nursing, 87362 Norwegian University of Science and Technology Faculty of Medicine and Health Sciences, Trondheim, Norway; 3 General Practice Research Unit, Department of Community Medicine, 60482 UiT The Arctic University of Norway Faculty of Health Sciences, Tromsø, Norway

**Keywords:** ethnography, Haiti, idioms of resilience, participatory research, resilience, youth

## Abstract

Haitian expressions of resilience also hold deep knowledge of human vulnerability. This longitudinal, qualitative study with young Haitians from urban shantytowns combines ethnographic and participatory methods to explore the complexities behind such idioms. Artistic and creative products made by or with the youth facilitated interviews, focus group discussions, and workshops. Through the life stories of participants and rich ethnographic material, this study presents locally situated idioms of resilience (and distress). By including local social ecology, the idioms were framed as historically and culturally rooted, thus shaping contextual, pragmatic, and gendered coping strategies grounded in embodied experiences of vulnerability and resistance. The study adds essential insights into Haitian resilience, revealing the local logics behind seemingly paradoxical statements. By drafting a conceptual framework for further studies on idioms of resilience, the study also makes a theoretical contribution to international resilience research.

## Introduction

In 2012, during my (the first author's) first fieldwork in Delmas, a suburb of the Haitian capital Port au Prince, I got to know 27-year-old Kenley (pseudonym^1^), who became a friend and cultural interpreter for me. He was also a colleague in the youth club I am reporting on here, recruited as a youth leader for his many talents: an acclaimed hip-hop musician and movie maker. He was also fluent in English, which he had taught himself by watching English TV shows downloaded from various free WiFi spots around town, on an old laptop he had got from his teacher, who had seen his potential. With all these skills, Kenley soon became an attractive employee, and especially a highly valued leader by the youth attending his classes.

As long as I have known Kenley, he has leant on a type of wisdom that I have heard presented repeatedly, in various forms. Kenley's version was: “I base my life on sadness. (…) You don’t understand. (…) I need it to feel happy.” The complexity inherent in Kenley's quote intrigued me, and his further explanation opened my eyes. Although he kept reaching his goals, he was still reminded of the seemingly never-ending social crisis around him.

Waking up to a soundscape of roosters, pigs, babies, and car brakes reminded Kenley of the extreme population density of his neighborhood, one of Delmas’ many scattered squatter towns, consisting of a blend of occupied, mainly half-finished concrete buildings and improvised buildings of mud and metal sheets. These neighborhoods are locally referred to as *getos* (ghettos), due to their exclusion from state support. In Delmas these *geto*s are scattered in between fenced-in properties of wealthy people, providing reminders of Haiti's enormous socio-economic divide. Kissing his mom goodbye reminded him of his father who was never there, how his mother had fought to feed and care for him, and how he now was the father figure for his two younger siblings. He made his way out of the *geto* through the maze of tracks and layers of intense smells of urine, jasmine flowers, adrenalin sweat, and pollution, all crystallizing like dust on his skin and reminders of the many surrounding health threats and lack of access to basic state services like sanitation, health care, and security. He greeted his neighbors: a network of friends, allies, and family preparing their street sales, many of them children out of school, too many of his friends unable to live out their dreams and talents. In addition, many of them suffered from preventable and treatable diseases, reminding him to honor also those they had already lost to unnecessary deaths.

Jumping into a ramshackle *taptap* (local taxi-bus), heading to his hard-won formal job, he felt obliged to pay back to his community. Kenley's background from a deprived neighborhood and his own experiences with trauma and despair instilled him with a constant bodily perception of others’ suffering. Only by accepting the pervasive sadness, he insisted, could he feel happiness, comfort, and space to thrive. The complexity hidden behind Kenley's expression guided me to seek out the local logics behind other apparently paradoxical statements of coping. In this article I explore such idioms of *resilience*.

While I acknowledge the deeply social nature of resilience processes, individual perspectives are also highly relevant. Based on empirical insights into the everyday lives of a group of young Haitians, I will examine how they define and evaluate major life challenges, aiming to explore how they negotiate the cumulative amounts of personal, collective, and historical traumas. I seek to uncover complex and sometimes hidden sources of coping, critically investigate these, and thus broaden and even challenge conceptual perceptions of Haitian resilience, adding to the international debate on idioms of resilience.

### Resilience

Resilience is a widely studied concept in various fields. In psychology, resilience is related to a person's capacity to, and process of, adapting to adversity in positive (socially desirable) ways (Masten, 2014; Ungar, 2013; Werner, 1993). There has been growing criticism of the concept, in particular for its cultural insensitivity (Masten & Wright, 2010), and for holding individuals, not systems, accountable for their own suffering and despair (Mahdiani & Ungar, 2021; Theron, 2016). Furthermore, in contexts of poverty, Bracke (2016) argues that celebrating resilience is “cruel” if “promises of flourishing” (p. 64) are empty, especially when the resilience of an oppressed people is misused to maintain neo-colonial structures.

This study's interest in resilience arose from the young people's wish to discuss how they overcame major challenges in life. Their stories made me understand individual resilience similarly to Ungar (2013), who defined the concept through the premise of local “social ecologies.” Understanding an ecology includes the structural backdrop of history, economy, and politics, and the social patterns of local culture. An individual's resilience can only be understood as the capacity to adapt and thrive within their local environment: what is “positive” adaption thus becomes *relative*.

Using longitudinal ethnographic and participatory methods, this study seeks to critically analyze observed phenomena related to resilience, thus providing culturally sensitive understandings of contextually relative resilience.

### Idioms of resilience

The hardship and suffering described by Kenley is common to most Haitians and is locally referred to as *lamizè* (the misery). A local Caribbean idiom of distress (Massé, 2007), *lamizè* describes a complex of adversities, including historical, political, and personal problems (Farmer, 1996; Maternowska, 2006; Schuller, 2016). Such permanently burdening conditions, especially adverse childhood experiences, can engender toxic levels of distress and even lead to disease and premature death (Felitti et al., 1998; Kirkengen, 2001; McEwen, 2004). Adversities may, as Kenley implies, also be a driving force for developing resilience.

Recognizing that local idioms of distress connote socio-cultural worldviews vital to make sense of suffering has proven valuable to cross-cultural health work for decades (Kaiser et al., 2015; Nichter, 1981; Worthman, 2019). In a recent special issue of *Transcultural Psychiatry*, Kaiser and Weaver (2019) call for greater attention to further idioms of resilience, spoken and bodily or symbolic expressions of well-being and positive outcome. Regarding Haitians, Rahill et al. (2016) similarly called for future research on resilience to pay attention to local Haitian expressions, and their rich culture of proverbs.

Derivois et al. (2018) highlight a paradox among street children, where “the most vulnerable populations [scoring highest on PTSD and depression symptoms] in Haiti have the highest resilience scores” (p. 79). Consequently, they ask whether Haitian resilience might indicate social or cultural pathology. The authors consider the possibility of inadequacies inherent in the current tools for measuring aspects of resilience. Below I will provide contextual knowledge to elucidate this paradox.

### Haitians, resilience, and youth

Since slavery times, generations of Haitians have lived lives saturated with uncertainty and burdened by precarity (Beckett, 2020; Farmer, 1996; Maternowska, 2006). Farmer (1996) famously explained current social inequalities and institutionalized oppression of Haiti's black population as postcolonial, or indeed neo-colonial, structural violence, leaving Haiti as one of the least developed nations in the world (Roser, 2013).^2^ In addition to social insecurity, natural catastrophes take lives almost yearly, the deadliest being the 2010 earthquake, which killed about 250,000 people. In one of the first studies of Haitian resilience, Nicolas et al. (2010) used Haitian phrases to illustrate the deep knowledge and experience of how to cope with natural disasters. Criticizing the international aid industry for bypassing local experience and personnel, Schuller et al. (2019) portrayed resourceful community responses to major natural catastrophes.

Research on Haitian resilience is growing. Derivois et al.’s (2020) extensive work has used locally adjusted questionnaires and scales, measuring resilience scores, to define so-called determinants of resilience.^3^ Others have investigated potential sources and expressions of resilience, such as the interview-based studies of Rahill et al. (2016), Kaiser and Fils-Aimé's (2019) ethnographic study of the use of traditional religious meaning-making to remove blame from individuals, or the study by Karray et al. (2016) that facilitated expressions of trajectories for survival and hope among street children through art workshops and interviews. Common to these researchers is their critical remarks on the leading Western definitions of resilience, and the need to use Haiti's remarkably complex history as a basis to understand contextually relevant sources of coping. Acknowledging this, the current study will explore the participants’ resilience processes, looking for local logics behind seemingly paradoxical statements or situations.

Rahill et al. (2016) and Joos (2017) found the societal structure of *lakou*s (literally “backyard”) to be an indicator of successful coping among urban earthquake survivors. Traditionally, local peasant society was organized in groups of relatives residing around a common backyard, creating independent small economies by swapping products and favors.^4^ This culture of reciprocity is still a central coping strategy in the urban *geto*, although strangled by displacement and economic inflation (Joos, 2017; Rahill et al., 2016).

The challenges of growing up in Delmas’ *geto*s are both context-specific and comparable to global trends. With 70% of the population aged under 30, Haiti's scarce resources are stretched (Daumerie & Hardee, 2010). Although most children now enroll in primary school, only 2% complete secondary education (Suzata, 2011). Thus, educated youth bear the expectation and responsibility to pursue higher education, find professional employment, and lift both themselves and their families out of poverty (Legha & Solages, 2015). However, among youth from urban shantytowns, unemployment rates approach 100% (Daumerie & Hardee, 2010).

It is central to literature within youth research to understand young people's *agency* in relation to societal, cultural, and structural limitations. In her influential paper, Bucholtz (2002) approached young people's experiences, positions, and practices in “untenable situations” from their *own* perspectives, thus unfolding social and creative ways for resistance and innovation (p. 535). In this article I will explore capacities for coping and agency development relative to the participants’ situations, searching for idioms of resilience in their everyday practice. I will show how their individual perspectives connect to local collective traditions and the broader social ecology.

## Methods

### Entry to the field

My entry to the field was through the Haitian-Norwegian NGO *Pwojè Ayïti*^5^ (Project Haiti), which offers low-cost education through a primary school, a program for women, and a free youth club. It is located between two busy roads with flourishing informal and formal shops and businesses. The area also holds numerous *ravins*, rivers of sewage, their banks densely populated by the socially deprived like Kenley's family and neighbors. Since 2007, I have frequently volunteered in Pwojè Ayïti's youth club, Etap Jènes (“Youth Stage”). Located in the NGO's facilities, it offers voluntary after-school activities for the local youth. Its main goal is to provide a free space where the youths themselves decide the content, shaped by available resources, including the competencies of local and international volunteers.^6^ Although the youth are given more responsibility in Etap Jènes than in the local community, the adult leaders of Pwojè Ayïti make major decisions.

In 2012, I recruited the study participants among current and previous members of Etap Jènes. Data were collected in 2012–2018 during 10 months of ethnographic fieldwork, thus gaining a longitudinal character. The first fieldwork period was four months, the next two months, and subsequent periods were 2–4 weeks. Although ethnographic participatory observation was the overarching method, different youth participatory action research (YPAR) sequences shaped each fieldwork period. When in Haiti, I shared an apartment with some of the youth leaders. Developing close relationships laid the foundation for the depth of my explorations. Living and learning their daily routines and passionate struggles for justice and stable lives informed my own life, both professionally and personally.

### Participants

Forty-one people, 18 girls and 23 boys, participated regularly in the research. They were aged 15–25 years at the study start. Participants were current and former leaders and members of the youth club, who volunteered to participate upon receiving invitations containing information about the study aims and methods. Those who declined were not asked to provide reasons. Consenting participants were repeatedly and explicitly informed of their right to withdraw. New participants could enter at any point.

About half of the participants attended Pwojè Ayïti's primary school, and many of their mothers were enrolled in Pwojè Ayïti's programs. Their parents were typically first-generation city dwellers, unemployed or street vendors. Most participants lived alongside the *ravin*s. A minority were lower middle class. All participants had completed primary school, while lack of funding made for irregular attendance at high school.

### Ethical considerations and choice of methods

The neocolonial reality of Haitian society implies an ethical obligation to address inequality and limited freedom affecting the participants (Madison, 2011). My own background illustrated such unfairness, contributing to major communication barriers, such as race, education level, and socio-economic status, in addition to language and culture. During the study period, I also became a medical doctor (although I did not practice as one for the youth). The biggest barrier was still that I, as an international volunteer, was associated with the leaders of Pwojè Ayïti, thus it took many years until the youths trusted my confidentiality.

This ethnographic study was therefore designed to facilitate young people's participation, leaning on YPAR and visual anthropology. YPAR aims to empower youth with skills for performing critical inquiry, taking action, and facilitating social change (Fine, 2012). Our explorations often sprang from art activities the youths were already learning in their youth club, to overcome the mentioned barriers (Conrad, 2004; Fine, 2012; Waage, 2013). The art produced offered powerful entrances to personal experience, often revealing new and embodied perspectives on a phenomenon (Liebenberg et al., 2014; Pink, 2007; Stoller, 1997). Visual anthropological methods thus guided our interpretation of the audio-visual material collected (Pink, 2007; Yonas et al., 2013).

The study was approved by the Norwegian Centre for Research Data (ID 34878). There was no local requirement of ethical approval for social science research in Haiti. To ensure the Haitian relevance of my ethical concerns, a group of local ethical advisers approved my plans. They were all coworkers of Pwojè Ayïti, with various fields of expertise, such as education, health, and security. They had contacts at local universities, whom they consulted for ethical advice on behalf of the study.

### Material and analysis

In accordance with the YPAR model, much of the data collection and analysis took place collaboratively, following a spiraling development typical of action research (Kemmis et al., 2013, p. 19). This spiraling co-production facilitated a mutual exchange of expertise between the participants and myself. I learned about their life and culture, while they acquired skills to lead project processes, which they then applied to the organization of their youth club.

This resulted in a diversity of material. Audio-recorded semi-structured interviews and focus group discussions were interpreted and re-interpreted between Creole and English and transcribed by me in English. Interpreters and participants were generally of the same sex. As my understanding of the language and culture improved, I managed increasingly without interpreters. Participatory observations and the numerous YPAR meetings were documented as field notes. Artwork such as songs, dances, plays, photos, films, or collaborative mapping (Clark, 2011) was documented by audio and video recordings. The material allowed for reflexive and explorative dialogues and interpretations regarding meaning and context. All transcribed texts were coded and categorized using qualitative content analysis (Malterud, 2012). The remaining material was analyzed thematically (cf. Clarke & Braun, 2017). Sub-themes were explored regarding overarching topics, which led to new spirals of action research activities. The findings were validated through YPAR sequences towards the end of the study. Although informed by findings from the larger study, the main sequences reported on here are:

1. In 2017, based on the advice of youth leaders and local ethical advisors, three girls and five boys were selected as representative of the various backgrounds in the larger youth group for semi-structured interviews. Using collaborative mapping, I explored their life stories, daily routines, and future plans.
2. Seemingly significant or paradoxical issues from these interviews, and expressions found through participatory observations, were then in 2018 reinterpreted through workshops with the whole youth group (36 participants), inviting them to explore the themes further through plays (Conrad, 2004) and collaborative mapping. Thus, my analysis and prioritization of capacities for resilience were challenged and finally approved by the participants.

In the following, I will present stories from three interviewees, chosen because they illuminate phenomena found relevant across the interviews and in the broader material. To provide anonymity, all names of participants are pseudonyms and stories are slightly changed or combined.

## Findings

A Haitian saying, *Naje pou soti*, holds key insights to understand local logics of resilience. Meaning “swim to exit,” it is often used by mothers to motivate their children. Paradoxically, very few people can swim, illustrating their difficulty in exiting poverty, indicating a need to be supported and kept afloat until one has learnt to “swim.”

However, the young participants in this study already had a sense of buoyancy. Insights into this were revealed during a workshop where the youth were challenged to arrive at what they considered the main individual qualities (*kalite*) needed to cope and stand a chance of reaching their life goals. After collaborative analysis and regrouping of these qualities, we arrived at four core categories, based on emic terms and explanations:

- Hope (*espwa*). Defined by the youth as including determination, patience, and hard work, closely linked to belief in God: “Without hope, there is nothing.”
- Pride (*fyèt*). Defined as self-esteem, integrity, and linked to morality: “Whatever you have to do, make yourself proud.”^7^
- Empathy (*senpati/sansiblite*).^8^ Defined as collectivity, awareness of vulnerability: “When you love and respect people.”
- Wisdom (*sajès*). Defined as contextual knowledge, locally and practically based: “Putting what you learnt into something in real life.”^9^

I will now illustrate how three participants, Dimitry, Marion, and Arvel, cleverly found different, gendered pathways to hope, pride, and empathy. Their stories portray local knowledge on how to avoid drowning, to cope and maybe even to thrive when growing up in Haiti's shantytowns.

### Negotiating a moral self: Dimitry's story

When Dimitry was three, his father's sudden death threw his family into deep poverty. Dimitry soon had to contribute to the household, and from age 13 he had to finance his own education. At the time of the earthquake, Dimitry's home collapsed. He saved himself and several neighbors out of the ruins, but did not manage to safe his youngest brother, whom he was babysitting that day. His mother blamed him for his brother's death and threw him out on the streets for about a year, where Dimitry, like other children and youth, found shelter in the many abandoned ruins. He witnessed murders and learnt the brutal moralities of the streets, where mere survival steered choices. He insisted on not selling drugs himself, but partly lived off the income of his friends who did.

Dimitry was 25 when I interviewed him, married with a son. Trying to support his family left no time, money, or energy to complete his final school year; he quit participation in Etap Jènes when his wife became pregnant. Steering away from jobs involved in corruption or crimes, he worked as a street vendor like his mother, and was frustrated at having applied in vain for several formal jobs:

I’m young, I’m strong, I can work. (…) The things I can do today, I can’t do when I’m 30. My legs will be weak and not last as long. (…) “*Ou vyeyi bone*” [You age early] – because of suffering. You work hard, you don’t eat well, you don’t sleep well, and at 25 you feel like 50.

Aware of the low life expectancy in Haiti and the low chances to exit poverty, he felt time was running out for him. However, Dimitry would not give up. He knew he was qualified for those formal jobs he sought. He knew the structures that kept him stuck in a difficult situation. Understanding this had taken much reflection, much of it done *nan tèt*, in his head. Since learning how to write at age 11, he had reflected on his life in diaries, later also in hour-long prayers. Whenever he felt frustrated or mistreated, he scrutinized his emotions and thoughts, reflecting on whether he experienced unfairness or fairness. This had given him the skill and strength to live a life guided by what he called *mowalite* – morality. He knew he was worthy of the stable life he sought. This was how he managed to accept his unfair situation and even feel proud of his actual achievements. Dimitry stated that if he did not find a well-paid job, he would be happy to contribute to his community by advising younger people.

Ideally, the youth explained, they should not be involved in morally dubious activities like corruption or crime, as this would endanger God's support. However, local Catholic morality is adjusted to the contextual desperation. Dimitry explained how his background and diary notes had taught him to always view behavior contextually and empathically: “It's not what people did or said, but why. ‘*Toujou mande poukisa*’ [always ask why].” Living a morally defensible life provides self-pride and is key to a salient resource for strength and hope: God's love and support. As mentioned, the youth viewed hope as essential to their very existence. Without hope they struggled to find meaning in life.

Dimitry's mixed moralities resonate with other participants’ statements and concur with local religious practices. Local religious belief systems are syncretized from Christianity (mainly Catholicism and Protestantism) and the spiritual *Vodou* religion (Desmangles, 1992, pp. 170–183). While Vodou practices are considered demonic by the Christian churches, many Vodou concepts/beliefs are taken for granted as reality, also by local priests and pastors.^10^ The participants belonged to different congregations and Christian religions, and one of them was a Vodouist. They all adapted their religious practices to circumstances, mainly realizing their relationship with God/Vodou *lwa*s (spirits) through moral behavior. They explained that their fluctuating religious practice often led to conflict with the parental generation. Their *bricolage*^11^ of moralities and religious practice reflects the complexity of local logics.

### Identifying as an advisor: Marion's story

When Marion was two, her parents abandoned her and her baby brother. Marion's grandmother raised her and paid her school fees for nine years. However, when Marion's aunt became head of the household, Marion and her brother had to leave school and lived as “*restavèks*” (child house slaves) (Restavèk Freedom, 2011). When 14, she escaped by moving to live with relatives. Although again keeping their house for her living, she was treated more respectfully and could even save money to start selling sandals, earning enough to continue school four years later.

Marion, now 24, still gave her brother food or money, but worried because he seemed depressed and was frequently on drugs. The siblings struggled to accept that their parents never seemed to care about them, evoking anger and painful thoughts and feelings. Marion said: “I always think, think, think about it. That's why I always have a headache.” Her brother, she said, even keeps asking: “Why don’t we die? Our mother and father are alive, but they don’t care about us, so why …?”

However, reflecting upon her life, Marion felt that thinking about her struggles had been useful. Her aunt's bad treatment taught her care and empathy, understanding that her aunt also suffered from the harsh realities, similar to Dimitry insisting on asking why. Even when receiving less food than the other children, Marion would always share. She dreamt of becoming a professional helper, such as a physician, diplomat, nurse, or “someone helping poor people.” Being a good advisor was her preferred goal.

The other interviewees also identified themselves as future consultants or helpers, saying: “*Mwen se yon konseye*” (I’m an advisor). This phrase is linguistically significant, as *mwen* (I) followed by *se* (to be, here: am) indicates ownership of the role as a *konseye* (advisor), just as one would declare a profession or nationality. For some, this might have reflected their roles in the youth club, but they related it more to their position in their neighborhoods. Being in school, they felt morally obliged to share their knowledge; but providing valuable advice also gave them a significant social position.

Many participants reported *reflechi twòp* “thinking too much,” which is an idiom studied across cultures, in Haiti by Kaiser and colleagues (2015), and found to be a common sign of mental health problems. Marion saw her excessive thinking as mentally burdening, although useful for empathically understanding others. Thus, identifying as an advisor represented personal growth.

Marion's story speaks of strictly gendered opportunities. She would not dare to seek support on the street, as the chances of being raped or abused are higher for girls. The chances of making a career within the system of local street gangs (called *baz*) are reserved for young men (Kivland, 2014). Accordingly, more young men suffer from street-based risks, like drug abuse or violence.

### Guarding one's secrets: Arvel's story

Arvel, 18 years old, lived in one room with his mother, two brothers, and one sister, whose different fathers contributed very little to the household. Some years ago, Arvel was pushed from behind under a passing truck. Bleeding and in agony, he faced a stranger pointing a gun at him, mimicking a shot, and kicking him before leaving him wounded on the ground. A friend took him to hospital where he had several operations free of charge due to special funding linked to the 2010 earthquake, and he gradually recovered. He told a cover story to people commenting on his scars and turned his limping into a ‘cool,’ hip-hop-like way of walking.

Arvel's secrecy about his traumatic experience was necessary for resilience. His physical scars, added to the psycho-social *lamizè*, made him particularly vulnerable to pity: “If people knew, they’d start asking about it. It’d make me start thinking about the accident. I’d be sad and maybe think about it the rest of the day.”

Arvel also realized that if the incident became publicly known, it would trigger rumors of him being either a gang member, which would endanger his reputation as a conscientious student, or haunted by spirits, jinxed (often by someone) with bad luck (see also Kaiser & Fils-Aimé, 2019). An old Haitian proverb says *Lè ou an devenn, lèt kaye kase tèt ou* (When you are jinxed, even curdled milk will break your head) (Jeanty & Brown, 1996), or, as Arvel interpreted it: “… anything can happen to you.” Either way, Arvel's nearest relatives would be endangered and he could be socially excluded. Consequently, he guarded his secret, saying he “keep(s) it ‘*nan tèt mwen*’ [in my head].”

Arvel, like Dimitry and Marion, managed to redefine his misfortune into useful experience, aiming for a career as a police officer or soldier, and already trying to advise others through his inspirational hip-hop lyrics.

During the semi-structured interviews, seven out of eight told something they termed their secret. Secrecy seemed vital when protecting their sources of hope, pride, and empathy from the self-centeredness of pain, suffering, and shame. They name this strategy *m’ap konsilte tèt mwen* (I consult my own head). *Tèt mwen* denotes both ‘my head’ and ‘myself,’ rendering the *tèt* (head) fundamental to selfhood and personal strength. As I will return to, showing capacity to move on from traumatic experiences might be viewed as a moral stance, helping their social network to focus on hope rather than despair.

Secrecy is highly gendered. While Arvel's secrecy protected him against rumors of being connected to the streets, Marion was secretive about her loneliness as an orphan, as it would not only attract pity but also render her vulnerable to sexual abuse.

Although secrecy was mostly not a choice, the youth were aware of the implicit threat of keeping secrets, linking it to spinning thoughts, headaches, panic attacks, shortness of breath, and “trouble up to our throats,” a perception of being strangled or choked.^12^ They repeatedly emphasized the urgent need for local therapists, with similar backgrounds to themselves, although not too local for fear of rumors. However, the young Haitians insisted their priority of consulting “their own heads” was also important, a pragmatic choice that developed their capacity for self-reflection and pride.

### Learning how to swim at the intersection of hope, pride, and empathy

The stories of Dimitry, Marion, and Arvel demonstrate the dangers of growing up in Haiti. Although their stories are embedded in tales of *lamizè*, the youth insisted on agency, and learnt through practice, interactions, and shared experiences with their family, friends, elders, or community leaders. As Kenley explained in the introduction, the youth continuously practice and embody their sense of hope, pride, wisdom, and empathy while living amidst unfairly distributed resources, experiencing daily racist, classist, or sexist comments. In this section I will emphasize findings from the broader material, observations, and group discussions, illustrating how the youth developed and practiced this embodied sense of agency. These social practices unfold local logics in apparent paradoxes.

When learning to “swim,” the participants must navigate the rough waters of poverty. With synergistic effects, hope keeps them afloat, while pride enables advancement. Their empathic bonds to others provide lifelines and respite. Although the youth might appear to be about to drown, life in the shantytowns of Delmas is more than mere survival. Despite dramatic experiences, everyday life, as I have observed it, has a clear sense of normality. This might be hard to recognize for an outsider.

The youth's idioms of resilience unfolded most clearly when they had to navigate the generational gap to their parents. Being the first literates in their families brought them closer to other social classes. Carefully building a socially respectable personality, the youth had to avoid judgment by their parental generation, while also finding creative, even innovative paths to stability.

When exploring the development of agency among Haitians, understanding the notion of respect (*respè*), and its central place in social life, is essential. One participant stated, receiving unanimous agreement: “As everybody knows, if you don’t have *respè* [respect], you’ll be nothing” (from workshop). Kivland (2014) explains that *respè* among male gang members means “feeling good” and spreading “a good feeling.” In a mapping exercise, the youth linked self-respect to their capacity for hope, pride, and empathy, underlining a need to *radiate* this positive attitude to receive respect from their community. Importantly, this had to be done in socially sensible ways, due to the threat of envy from others and enemy-making.^13^

This practice showed during the popular pastimes of *pale kaka* (talking ‘shit’ or nonsense) and *bay blag* (telling jokes). Humor was an outlet for frustration and emotionally burdening experiences. Although not explicitly revealing secrets, they turned personal experiences into general funny stories, based on their knowledge of what to share, how, and with whom. This gave them veiled, often gender-divided outlets for secrets. Young men often *pale kaka* over some beers at a street cafe in the evening, while young women mainly *pale kaka* within the safety of four walls and fewer pairs of ears.

Humor was a core of local social life. Jokes and funny stories, self-irony, friendly bullying, and satirical criticism of the state and societal structures were ingrained in most conversations, evoking loud and heartfelt laughter. The culture of ridiculing social misery and structural injustice provided a sense of empathic belonging and an opportunity to translate negative experiences and emotions into a constructive desire for change in one's future and in society. Telling jokes thus becomes a respectful, empathic contribution to the common good, by making people laugh and ‘feel good,’ as well as through the exercise of resistance against oppressive structures. The participants’ self-respect was developed socially and nourished by self-reflection.

Such embodied idioms of resilience were easy to spot during the participants’ weekly traditional drum and dance class. As they played the traditional rhythms and performed the steps of warrior dances inherited from their ancestors who gained their country's independence, there was no room for shyness or disturbing thoughts about today's *lamizè*. The inherent power persisted even after class, typically leading to talk about ancestral pride and a common source of hope: the possibility of a new revolution bringing justice to their country and people. The youth would comment that such a revolution starts with self-respect, demonstrating this bodily as a lifted head and a strong and erect body posture. To a large extent, this was their everyday body posture.

The youth's drum and dance sessions, tutored by respected local artists, helped them relate to their ancestors and their historical roots. The sessions provided spiritual and bodily experiences which enhanced their embodied pride, enabling them to stand erect despite oppression and inequity. Vodou*-*related practices are generally abandoned by the urban parental generation. Young people are thus polarized between pride in their ancestors’ achievements and shame arising from the contested practices. Young Haitians are starting to trust their cultural sources of pride and hope, hoping to achieve change for themselves and coming generations.

Some youth, like Kenley, had found ways out. This depended on stable reciprocal bonds to others, and on expanding their social network by crossing social classes, through friendships, scholarships, or marriage. Since my last fieldwork in 2018, Mariel had earned enough money from selling sandals to start her final high school year, but the next school years were cancelled due to the 2019 political protests,^14^ followed by COVID-19 lockdowns. She still saves her money. Arvel has had success as a hip-hop musician, his group getting national attention. He hopes to earn money to finish high school from music and movie-making. Dimitry has worked as an English interpreter, although he and his wife are still street vendors. Unlike their parents’ generation, they have access to family planning, and are postponing having another child until they can afford it.

Although many participants wanted to leave Haiti for a break or to study, their wish to return and contribute to their people was univocal. They refused to accept the unjust structures of their society but sought change for the better, speaking of a non-violent revolution. Combining the wisdom from shantytown life with university degrees, especially the older youth spoke of changing the system from within, liberating their minds from shame and despair first.^15^ With their embodied sense of life's vulnerability combined with their empathic bonds to family and neighbors, they spoke of a social obligation to keep searching for ways to change the status quo, also finding strength from a growing sense of spiritual connectedness to their revolutionary forefathers. In late 2021, the youth expressed despair after a tough fall with an anarchic situation in the streets following the assassination of Haitian President Moïse.^16^ Many had spent the fall helping out after the August 14th earthquake in southern rural Haiti. As Dimitry stated, in times of resignation they can still advise others.

Their steadfast confidence in themselves, and their hopes of achieving the impossible, imbues the paradox of *naje pou soti* with local logic. Learning how to swim in the context of Haiti's shantytowns implies clever and pragmatic social positioning, consistent with the local reciprocal traditions, as well as with the *lamizè* and uncertainty surrounding them. The youth were already swimming, but the fog of structural violence might just hinder them from finding a way out.

## Discussion

Our stepwise collaborative analysis revealed seemingly paradoxical expressions as complex sources of resilience, interwoven with sources of distress. The young participants showed a capacity to redefine negative experiences as experience to learn and grow from. Entwined with capacities for developing hopeful, proud, and empathic identities, this network of protective capacities was developed from a wide range of sources of local wisdom: historical, traditional, religious, and ancestral/spiritual, merged with contemporary knowledge from school, the youth club, *baz* culture, and the Internet, forming what the youth term practical wisdom. Embodying and radiating hope, pride, empathy, and wisdom was protective, earning respect and building their self-respect.

Through the complex interplay between suffering and well-being, the youth incorporated profound knowledge of *lamizè* into their striving for agency. With the local, transgenerational knowledge of life's uncertainty as a basis, they could find pragmatic sources of resilience. Resilience among Haitians does not come without despair (Rahill et al., 2016). The youth's identity-building involved wrestling with shame and despair, carefully and cleverly practicing their capacity for hope, pride, and empathy, developing local, socio-cultural, and gendered pathways towards inner stability.

Our findings align with Ungar (2013) and his argument that “resilience can look like pathological adaptation” (p. 262) if researchers do not engage in understanding young people's clever navigations of threatening social environments. By contrast, our study challenges the suggestion of Haitian resilience as social pathology (Derivois et al., 2018) and nuances previous research on resilience among young people in Haiti, which has mainly been conducted among street-based children (Cénat et al., 2018; Derivois et al., 2020; Karray et al., 2016). These authors appreciate the complex historical, spiritual, and collective sources of coping and thriving. However, their analysis does not incorporate perspectives of resilience as relative to local social ecology.

My findings support Derivois et al.’s (2018) suggestion of resistance as a suitable term to describe the local capacity to endure rough times, patiently hoping for new opportunities. Bourbeau and Ryan (2018) argued that resistance can be a form of resilience, and that the two are not, as commonly viewed in resilience research, two mutually exclusive phenomena (e.g., Bracke, 2016). Most of the idioms of resilience uncovered in this study drew on Haiti's deep culture of resistance against surrounding oppressive structures.

This also answers Bracke's critique of the resilience concept. The study participants’ creative bricolage of traditional and contemporary, local, and international worldviews constructed meaningful and resilient ways of resistance, despite only a minute hope of change in their living conditions. Compared to their parents, however, their opportunities had expanded, their role as educated people led to respected positions, and their technological advances provided connectedness and participation in grassroots movements. Their constructive and creative bricolage of norms and morals is typical of youth (Bucholtz, 2002) and a common Caribbean practice (Knepper, 2006).

In response to Derivois et al. (2018), there certainly exist social environments forcing people to adapt in dangerous ways. In some situations, mere survival is already an accomplishment. The risk of “drowning” due to inhumane surrounding structures does not invalidate a young survivor's persistent capacity for resilience. Instead, it points out the need to support struggles for the liberty of the oppressed. The data in this study, unfolding complex, partly hidden local logics of thriving, underline the relative nature of resilience as a process towards improvement, or for many of the youth in this study, the process towards a stronger resistance to oppression. I would thus argue that pathological resilience does not exist.

Interestingly, to unfold the local logics behind many of the idioms of resilience presented by the youth, their spoken words were insufficient. Through persistently trying to understand their worldviews and observing how they held their bodies, I found an entrance to explore also their embodied experience. All participants have embodied adverse, even traumatic, experiences, but also constructive, nurturing experiences of coping and well-being. However, they intentionally choose to inform their bodily posture by nurturing hope, pride, and empathy. Accepting that despair, shame, and collective misery were parts of their lifeworld, they embodied an understanding of life's vulnerability.

This is illustrated by the implications of *respè* among the youth in this study. There is a synergistic effect between respecting others, being respected, and one's own development of self-respect, none of which can exist without an embodied understanding of other people's misery. Embodiment and bodily expressions have long been studied in anthropology (Csordas, 1990), unlocking important knowledge about transcultural idioms of disease (Kirmayer & Ramstead, 2017).

Kenley's initial statement of basing his life on sadness should be acknowledged as pragmatically embodied awareness of life's vulnerability, just as Panter-Brick (2015) argues that resilience, like hope, “is essentially the production of a coherent narrative that explains personal and collective experiences” (p. 240). His was an empathic standpoint that, dependent on the support he received back from the local community, enhanced his well-being and capacity to resist misery. Misunderstanding Kenley's statements as simply an expression of Haitian *lamizè* might endanger his and his peers’ possibilities for greater well-being. This study points out the risks of overlooking the local cultural, historical, political, and economic contexts and the embodied expressions of coping and thriving, especially the deep knowledge of oppression and shame contained in such coherent and resilient expressions. These findings add essential perspectives to Haitian and international resilience research, contributing conceptually to understand local idioms of resilience.

### Strengths and limitations

The longitudinal character of the study and the gradual process of familiarization with a group of deprived young Haitians enabled a body of authentic accounts. The richness of this material, representing a close-up of everyday struggles, fears, concerns, pains, pleasures, and achievements, led to insight into survival strategies invented and developed despite limited options.

This study has several limitations. Firstly, my initial unfamiliarity with the language and culture necessitated local interpreters during most of the project. Similarly, an unavoidable power imbalance inherent in my various roles had to be accounted for and reflected upon. As a European, female, well-educated and privileged researcher and, simultaneously, an activity leader, I was clearly advantaged. However, mutual trust and confidence gradually mitigated this discrepancy. There are also obvious limitations to the transferability of findings based on a relatively small group of youth, all connected to the same NGO. Not until the youth started criticizing their NGO and me did I start to trust my findings.

## Concluding remarks

Through dwelling on apparently paradoxical statements and behaviors among and with the participants, relevant local survival strategies and vital local sources of resilience were revealed. With the relative nature of resilience in mind, the resilience processes observed in this study, leaning on cultural traditions and contemporary knowledge, should be viewed as local normality, certainly not misunderstood as pathological.

Responding to Kaiser and Weaver (2019), who call for the development of a conceptual framework for studies of idioms of resilience, this study suggests firstly that cross-cultural research on idioms of resilience must integrate comprehensive exploration of local “social ecology,” uncovering the complex historical, economic, political, and socio-cultural backdrop of apparent paradoxes. This should include studying how seemingly paradoxical coping strategies may indicate a different logic than expected. Importantly, embodied experiences and their bodily expressions should be observed, with particular attention to apparent paradoxes. Lastly, sources of resilience can be intimate, hidden behind numerous communication barriers. I suggest enabling participant ownership of all research processes through participatory methods.

Although the study reveals a need for drastic and coordinated local, national, and international initiatives to release young Haitians from oppressive neocolonial structures, the young voices from Haiti describe a local, cultural wisdom that must not be ignored when assistance is offered. The study illustrates an urgent need to expand the debate in international resilience and trauma literature to include searching for and critically assessing idioms of resilience.

## Implications for further research

More longitudinal, ethnographic, participatory, and transdisciplinary research is needed on this topic, along with contributions from local researchers with a deep understanding of language and other cultural expressions, preferably with knowledge of lower-class life in Haiti. Researchers studying resilience anywhere in the world should search for local logics behind apparent paradoxes.

## Notes

1. Details of Kenley's background have been changed to provide anonymity.

2. Read more in Dubois (2012) and Schuller (2016).

3. The work of this milieu is too extensive to cite as a whole in this article.

4. Managing through these *lakou*s was seen as resistance to slave-like contracts in what had become state-owned plantations (Dubois, 2012, pp. 107–115).

5. A non-religious, non-political NGO, spelled *Prosjekt Haiti* in Norwegian; see www.prosjekthaiti.org.

6. Activities were offered in digital media, music, dance, sports, language, conflict resolution, project management, and leadership.

7. This fits with Tracy and Robins’ (2007) definition of pro-social pride, in contrast to hubristic pride which is linked to narcissism.

8. *Senpati* can be translated as both sympathy and empathy. The youth used it in the sense of empathy, also using the word *sansiblite* which translates to sensitivity.

9. As I will show throughout the article, *Sajès* is also used about religious, often biblical knowledge.

10. A deep analysis of how the different religious backgrounds shape the worldviews of the participants is outside the scope of this article. See e.g., Vonarx (2007) or Mocombe (2017).

11. *Bricolage* was first termed by Lévi-Strauss (1966), to describe the artistry of using anything at hand to solve a problem.

12. This could also be interpreted as signs of post-traumatic stress disorder (Cénat et al., 2018).

13. There is a considerable body of literature dealing with the intricate relationship between trust, secrecy, enemies, and sorcery in Haitian social life; see e.g., Kaiser and Fils-Aimé (2019).

14. The protests arose from the anti-corruption movement *Kote kob Petro Caribe’a?* (Where is the Caribbean Petrol money?), which uncovered the disappearance of US$2 billion of the Venezuelan Oil Program; see e.g., Nugent (2019).

15. Very much inspired by local artists and musicians; see e.g., Fleurant (2006).

16. See e.g., news articles by Abi-Habib (2021) and Montes (2021).
